# Herlyn-Werner-Wunderlich syndrome with urethrovaginal fistula: A rare case report

**DOI:** 10.1016/j.eucr.2021.101911

**Published:** 2021-10-25

**Authors:** Kuncoro Adi, Bambang S. Noegroho, Rani Septrina, RM Sonny Sasotya, Dikki Drajat K, Daniel Saputra

**Affiliations:** a Urology Department, Hasan Sadikin Academic Medical Center, Universitas Padjadjaran Bandung, Indonesia; b Plastic Surgery Department, Hasan Sadikin Academic Medical Center, Universitas Padjadjaran Bandung, Indonesia; c Obstetric and Gynecologic Department, Hasan Sadikin Academic Medical Center, Universitas Padjadjaran Bandung, Indonesia; d Pediatric Surgery Department, Hasan Sadikin Academic Medical Center, Universitas Padjadjaran Bandung, Indonesia

**Keywords:** Congenital, HWW syndrome, Menarche

## Abstract

Herlyn-Werner-Wunderlich (HWW) syndrome is difficult to diagnose. We report our first experience of fistula repair in HWW syndrome in Hasan Sadikin General Hospital Indonesia. A 12 years old girl presented with urinary retention, and was consulted to urology because the urethral meatus could not be found. MRI showed two separate uteri, cervices, and vaginas. Complete separation of vagina and urethra was done. After 2 years follow up, the patient had no complaint. HWW syndrome should be suspected in cases with pelvic pain or urinary retention during menarche period among teenagers and neonatal cases with any renal malformation.

## 1 Introduction

The Herlyn–Werner–Wunderlich (HWW) Syndrome is a rare variant of mullerian duct anomalies (MDA) characterized by the combination of uterus didelphys with obstructed hemivagina and ipsilateral renal agenesis.^1^ This syndrome first described in 1922. It is sometimes also referred to as obstructed hemivagina and ipsilateral renal anomaly (OHVIRA) syndrome.^1^, ^2^, ^3^

The true incidence of this anomaly is unknown, but it has been reported between 0.1% and 3.8%, and it is accompanied by unilateral renal agenesis in 43% of cases.^2^^,^^3^ The diagnosis usually discovered with non-specific symptoms, like increasing pelvic pain, dysmenorrhea and palpable mass due to the associated hematocolpos or hematometra, which results from retained, longstanding menstrual flow in the obstructed vagina.^4^^,^^5^

Here, we report a case of a teenage girl presented with urinary retention due to blood clot in urethra after menarche and was associated with an obstructed hemivagina, uterine didelphys, and agenesis left kidney.

## 2 Case presentation

A 12-year-old female patient came with urinary retention right after the patient had menstruation. Urology consultation was proceeded due to difficult urethral catheterization where urethral meatus could not be found by emergentologist. There was no urination complain before menstruation.

**Fig. 1:**
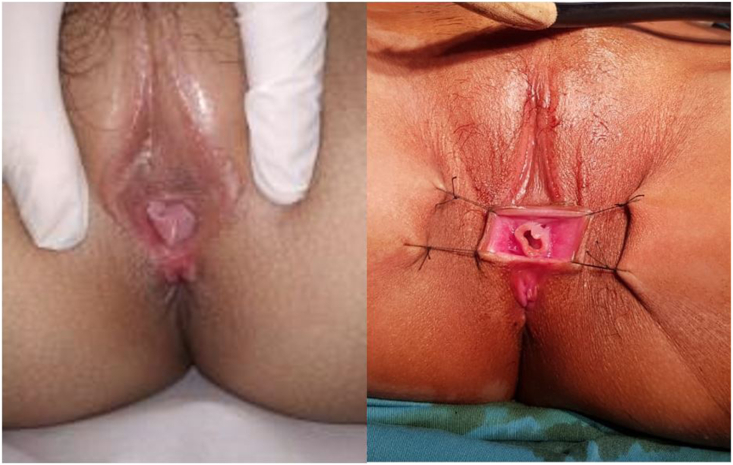
Clinical photograph of the perineal region showing a single opening atthe *introitus*.

On physical examination, we found only one visible ostium of which we suspected functioning as urethra and vagina, with both major and minor labia present (Fig. 1). The findings on urinalysis were cloudy color, erythrocyte sediments are over 50 and leukocyte sediments within 5–9. The chromosome analysis showed the karyotype result was 46 XX.

**Fig. 2:**
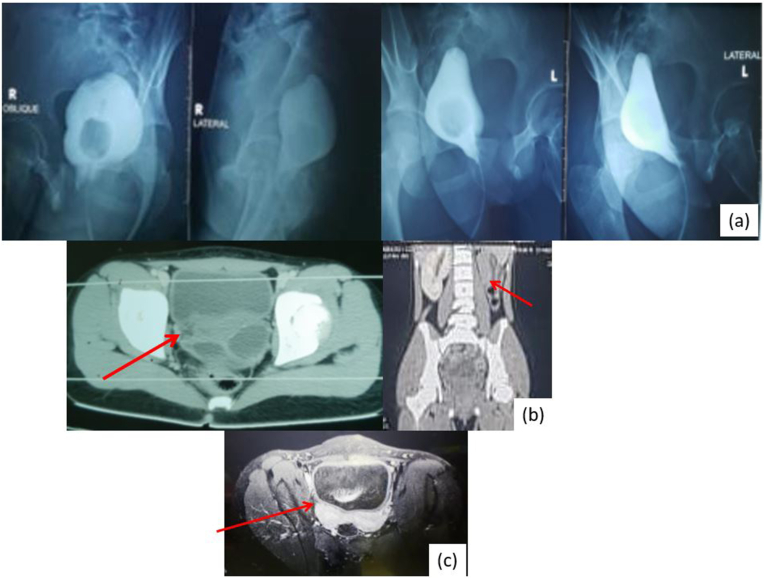
Radiologic Imaging of the patient a. Urethrocystography, b. MSCT of Abdomen and Pelvis, c. MRI Abdomen Pelvis.

Urethrocystography (fistulography) was performed; however, there was no fistula found (Fig. 2a). CT-Scan of the abdomen showed agenesis of left kidney, and uterus didelphys with hematocolpos (Fig. 2b, arrow). MRI of the abdomen and pelvis revealed two pear-shaped structures were noted in the pelvis suggestive of uterus didelphys bicolis. Fluid accumulation was found and suspected vaginal atresia with secondary hematometrocolpos (Fig. 2c, arrow).

**Fig. 3:**
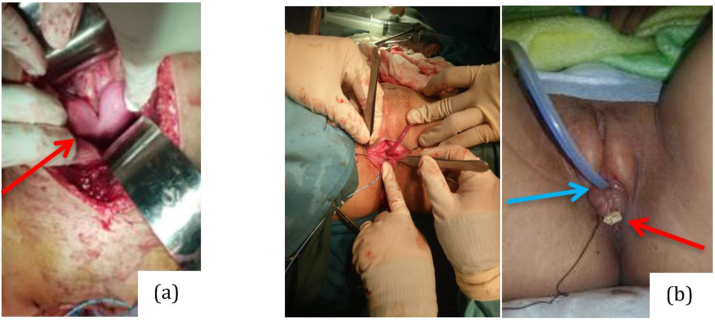
Operation and Result (a) Uterine Didelphys (arrow); (b) Separation channel for Urethra (blue arrow) and Vagina (red arrow). (For interpretation of the references to color in this figure legend, the reader is referred to the Web version of this article.)

Urethrocystoscopy revealed that there were 2 tracts about 2 cm from the ostium orifice connecting to vagina and bladder, and left ureter orifice could not be identified. From transpubic exploration was found uterine didelphys (Fig. 3a, arrow), double cervicals and vaginas. Muscle stimulation result was excellent (with API 0.21). The soft tissue of vagina and urethra was separated, then soft tissue around urethra was made for externalization of the urethra, the rest soft tissue was used for making vaginoplasty.

The surgery was successful with the results of 2 separate channels for urethra and vagina (Fig. 3b*).* After two years follow up, clinically, patient never had any complaints of urinary retention during menstruation, and her parents claimed that its appearance resembled to normal. There were mild symptoms from the IPSS score with a score of 7, and the patient was pleased with her quality of life. In order for further evaluation, the patient was scheduled to follow-up at Urology outpatient clinic annually.

## 3 Discussion

The overall prevalence of MDA is estimated to be 2%–3% among all women, with an incidence of 1/200–600 among fertile women. Hypoplasia and agenesis of the uterus and proximal vagina account for 5%–10% of Müllerian ducts anomalies, whereas didelphys uterus accounts for approximately 11% of Müllerian duct anomalies. Renal tract anomalies are associated with MDA in as many as 30% of cases. A complete or partial vaginal septum is present in 75% of women with didelphys uterus.^1^^,^^3^

Most patients are diagnosed during the menarche period. Determining an accurate diagnosis is challenging since menstruation occurs regularly sometimes; and when the patient complains of symptoms of cyclic dysmenorrhea, they are usually prescribed anti-inflammatory drugs and oral contraceptives, thus causing a delay in the diagnosis because of the reduced or eliminated menstruations**.** The potential complications of this syndrome are distinct in acute complications, such as pyohematocolpos, pyosalpinx, or pelviperitonitis, and long-term complications, such as endometriosis, pelvic adhesions, and increased risk of infertility.^4^^,^^5^

Ultrasonography is the diagnostic modality of choice in the detection of Müllerian duct anomalies because of the ubiquitous and minimally invasive nature of this imaging technique. However, MRI is the most accurate method for diagnosis by providing more detailed information regarding uterine morphology and continuity of each vaginal lumen with fluid content nature. It can also effectively depict the relationship between urogenital structures, the pelvic muscle complex, and associated spinal or renal abnormalities. Laparoscopy is not mandatory but could be helpful in confirming the diagnosis when the radiological imaging is inconclusive.^1^^,^^4^

The preferred surgical approach for patients with HWW syndrome is the reconstruction and marsupialization of the vaginal septum. Laparoscopy can also be therapeutic in some selected cases, such as drainage of hematocolpos or hematometrocolpos, septectomy, or marsupialization of the hemivagina.^1^^,^^5^

## 4 Conclusion

The diagnosis of this syndrome is difficult due to its rarity. A high index of suspicion is required for its diagnosis, especially if the external genitalia appear normal. Herlyn-Werner-Wunderlich syndrome should be suspected in cases with pelvic pain or urinary retention during menarche period among teenagers and also in neonatal cases with any renal malformation. Prompt diagnosis is based on clinical suspicion and timely intervention by a team of multiple expertise is essential for preventing potential complications.

## Consent

Written informed consent has been provided by the patient to have the case details and any accompanying images published, as stated by Committee on Publication Ethics (COPE) Guidelines, for the benefit of developing educational sciences. Institutional Approval is not required to publish this case.

## Source of support

None.

## Declaration of competing interest

The authors declare that they have no financial connections with any companies of relevance for this article.
